# Supplementary material for: Inflammatory pathways amongst people living with HIV in Malawi differ according to socioeconomic status
Source: PLoS One. 2021 Aug 25;16(8):e0256576. doi: 10.1371/journal.pone.0256576 (PMC8386842; doi:10.1371/journal.pone.0256576)
Supplement: S1 File — (DOCX) [file pone.0256576.s001.docx]

**Socioeconomic risk factors for immune activation differ according to inflammatory pathway amongst Malawian adults presenting with HIV: Supplementary Material**

S1 Table: Median plasma biomarker values according to socioeconomic variable for all participants (PLWH and HIV negative)

| **Socio-economic variable** | | **n** | **IL1Ra** | **sCD163** | **SAA** | **CRP** | **sVCAM** | **sICAM1** | **MIP1β** | **IP10** | **MCP1** | **bFGF** | **VEGF** | **IL7** | **IFNγ** | **IL1β** | **IL2** | **IL4** | **IL6** | **IL8** | **IL10** | **IL12p70** | **IL13** | **TNFα** |
| --- | --- | --- | --- | --- | --- | --- | --- | --- | --- | --- | --- | --- | --- | --- | --- | --- | --- | --- | --- | --- | --- | --- | --- | --- |
| **Roof** | **Grass** | 21 | 228.2 | 876,752.1 | 2,006,362.2 | 2,695,435.8 | 673,871.2 | 594,157.7 | 58.7 | 801.6 | 86.0 | 9.9 | 82.3 | 10.7 | 9.7 | 0.5 | 0.6 | 0.0 | 0.8 | 8.7 | 1.1 | 0.3 | 1.0 | 6.5 |
|  | **Corrugated** | 338 | 285.4 | 688,292.8 | 2,355,399.5 | 4,307,845.6 | 545,061.5 | 587,289.7 | 74.1 | 730.5 | 76.1 | 14.5 | 94.1 | 10.0 | 9.1 | 0.3 | 0.5 | 0.0 | 1.2 | 4.7 | 1.1 | 0.2 | 1.0 | 6.4 |
|  | **Other** | 9 | 251.2 | 505,352.6 | 2,836,981.7 | 2,302,071.5 | 341,268.7 | 545,441.1 | 40.2 | 506.6 | 65.8 | 22.3 | 74.4 | 11.0 | 5.0 | 0.2 | 0.4 | 0.0 | 1.0 | 5.3 | 1.2 | 0.1 | 1.1 | 3.5 |
| **Walls** | **Sundried brick** | 167 | 275.9 | 689,835.4 | 1,716,557.6 | 3,102,335.3 | 547,286.1 | 594,894.5 | 76.8 | 585.2 | 74.4 | 10.9 | 86.4 | 10.4 | 8.1 | 0.3 | 0.5 | 0.0 | 1.1 | 4.4 | 1.0 | 0.2 | 1.0 | 6.5 |
|  | **Burnt brick** | 180 | 295.8 | 689,659.7 | 3,216,917.9 | 5,086,058.1 | 527,760.4 | 580,252.8 | 72.7 | 758.8 | 75.9 | 15.9 | 95.8 | 9.9 | 8.5 | 0.3 | 0.5 | 0.0 | 1.2 | 5.3 | 1.1 | 0.2 | 1.1 | 6.2 |
|  | **Other** | 20 | 292.6 | 715,645.5 | 2,525,209.8 | 5,806,829.8 | 497,026.7 | 543,960.7 | 66.5 | 893.6 | 81.6 | 43.3 | 131.6 | 11.5 | 14.9 | 0.1 | 0.7 | 0.0 | 1.2 | 6.9 | 1.4 | 0.2 | 0.7 | 6.6 |
| **Floor** | **Earth** | 55 | 246.8 | 699,638.2 | 2,237,447.2 | 2,477,268.4 | 555,465.2 | 562,391.7 | 86.2 | 768.6 | 72.8 | 16.6 | 82.2 | 11.4 | 8.0 | 0.2 | 0.5 | 0.0 | 0.8 | 3.8 | 1.3 | 0.2 | 1.1 | 5.9 |
|  | **Brick** | 8 | 381.5 | 955,635.2 | 6,430,564.9 | 6,692,766.4 | 588,942.7 | 647,141.8 | 76.0 | 439.6 | 74.6 | 29.5 | 57.6 | 6.1 | 6.3 | 0.5 | 0.5 | 0.0 | 1.2 | 3.4 | 0.9 | 0.3 | 0.9 | 7.3 |
|  | **Cement** | 290 | 286.6 | 677,854.2 | 2,079,878.4 | 4,143,970.7 | 528,850.8 | 581,710.1 | 71.6 | 674.0 | 77.8 | 13.6 | 99.0 | 10.0 | 8.8 | 0.3 | 0.5 | 0.0 | 1.2 | 5.2 | 1.1 | 0.2 | 1.0 | 6.1 |
| **Tenure** | **Bought** | 35 | 235.4 | 797,174.0 | 2,684,191.3 | 2,526,275.9 | 441,028.4 | 544,875.0 | 76.7 | 767.4 | 63.4 | 18.4 | 142.6 | 12.0 | 10.5 | 0.2 | 0.5 | 0.0 | 1.1 | 5.5 | 1.0 | 0.2 | 0.9 | 5.8 |
|  | **Built** | 103 | 299.4 | 670,655.9 | 2,185,047.3 | 3,592,466.1 | 544,040.4 | 595,455.9 | 71.5 | 751.2 | 76.8 | 10.0 | 93.8 | 9.3 | 9.7 | 0.2 | 0.6 | 0.0 | 1.2 | 4.9 | 1.0 | 0.2 | 1.1 | 5.6 |
|  | **Renting** | 191 | 276.3 | 674,795.6 | 2,355,399.5 | 3,883,748.6 | 547,135.7 | 581,137.9 | 71.8 | 678.2 | 78.0 | 15.7 | 85.1 | 10.7 | 7.8 | 0.3 | 0.5 | 0.0 | 1.0 | 5.0 | 1.2 | 0.2 | 1.0 | 6.5 |
|  | **Relatives** | 21 | 348.3 | 723,988.2 | 1,019,217.8 | 5,163,789.7 | 573,102.6 | 572,946.7 | 72.8 | 568.3 | 78.3 | 16.7 | 96.3 | 6.2 | 8.3 | 0.7 | 0.5 | 0.0 | 1.4 | 9.3 | 1.0 | 0.2 | 2.2 | 6.8 |
| **Toilet** | **Flush** | 55 | 298.1 | 715,645.5 | 2,267,527.5 | 5,597,268.0 | 551,165.0 | 553,300.1 | 76.0 | 775.1 | 76.1 | 29.9 | 110.1 | 9.5 | 6.6 | 0.3 | 0.4 | 0.0 | 1.2 | 5.1 | 1.1 | 0.2 | 0.9 | 5.2 |
|  | **Latrine** | 316 | 278.0 | 688,497.1 | 2,260,691.6 | 4,008,697.0 | 544,147.1 | 587,289.7 | 72.7 | 691.7 | 75.5 | 13.6 | 91.4 | 10.1 | 9.7 | 0.3 | 0.5 | 0.0 | 1.1 | 5.1 | 1.0 | 0.2 | 1.0 | 6.5 |
| **Number of bedrooms** | **1** | 28 | 247.0 | 791,648.6 | 3,490,302.8 | 3,162,584.0 | 520,352.5 | 551,961.7 | 59.2 | 431.3 | 77.2 | 11.0 | 64.5 | 5.9 | 6.1 | 0.4 | 0.5 | 0.0 | 1.5 | 6.0 | 0.9 | 0.2 | 1.0 | 6.4 |
|  | **2** | 95 | 264.9 | 652,335.1 | 1,446,825.8 | 3,563,947.3 | 545,383.0 | 507,447.1 | 71.5 | 574.6 | 67.6 | 10.3 | 95.7 | 10.2 | 8.8 | 0.3 | 0.6 | 0.0 | 0.9 | 4.2 | 0.8 | 0.2 | 0.9 | 6.2 |
|  | **3** | 106 | 304.8 | 674,501.7 | 2,102,567.1 | 3,798,089.9 | 562,850.7 | 616,859.1 | 83.5 | 612.7 | 72.8 | 14.7 | 93.2 | 11.5 | 8.1 | 0.4 | 0.5 | 0.0 | 1.2 | 4.7 | 1.2 | 0.2 | 1.0 | 6.4 |
|  | **4** | 85 | 285.5 | 654,153.0 | 3,469,252.4 | 7,088,253.0 | 537,172.5 | 605,974.6 | 72.6 | 829.5 | 86.4 | 14.3 | 89.9 | 9.9 | 10.2 | 0.3 | 0.6 | 0.0 | 1.3 | 6.5 | 1.2 | 0.2 | 0.9 | 6.4 |
|  | **5** | 29 | 269.9 | 808,329.7 | 2,200,921.5 | 5,250,358.7 | 519,264.4 | 530,396.2 | 67.6 | 807.4 | 70.4 | 15.8 | 112.3 | 8.1 | 11.7 | 0.2 | 0.5 | 0.0 | 1.1 | 3.7 | 0.7 | 0.2 | 1.6 | 6.6 |
|  | **>5** | 29 | 305.7 | 858,889.9 | 2,980,046.6 | 3,044,893.7 | 525,487.4 | 592,437.3 | 75.4 | 788.5 | 90.9 | 22.2 | 103.2 | 10.9 | 8.2 | 0.1 | 0.5 | 0.0 | 2.2 | 5.2 | 1.2 | 0.2 | 1.1 | 6.1 |
| **Kitchen as a separate room** | **No** | 241 | 278.0 | 804,448.4 | 1,734,457.8 | 2,758,471.5 | 547,135.7 | 547,467.9 | 76.6 | 658.3 | 72.4 | 15.8 | 87.0 | 9.2 | 8.9 | 0.3 | 0.5 | 0.0 | 1.1 | 4.3 | 1.1 | 0.2 | 1.0 | 6.3 |
|  | **Yes** | 127 | 286.6 | 654,153.0 | 3,070,050.5 | 5,086,058.1 | 544,663.9 | 595,455.9 | 71.7 | 744.0 | 77.9 | 14.5 | 92.2 | 10.2 | 9.1 | 0.2 | 0.5 | 0.0 | 1.2 | 5.5 | 1.1 | 0.2 | 1.0 | 6.4 |
| **Electricity** | **No** | 167 | 260.1 | 694,736.8 | 2,457,045.5 | 3,847,899.6 | 566,542.0 | 593,638.2 | 78.7 | 612.7 | 74.8 | 14.7 | 88.6 | 9.9 | 8.0 | 0.3 | 0.5 | 0.0 | 1.1 | 5.3 | 1.0 | 0.2 | 1.1 | 6.1 |
|  | **Yes** | 201 | 302.2 | 692,735.1 | 2,062,833.1 | 4,466,693.5 | 511,277.2 | 578,707.4 | 70.5 | 766.0 | 76.1 | 14.5 | 100.3 | 10.2 | 10.3 | 0.3 | 0.5 | 0.0 | 1.2 | 5.0 | 1.1 | 0.2 | 0.9 | 6.7 |
| **Water** | **Private domestic tap** | 95 | 273.2 | 783,438.9 | 1,636,164.0 | 2,120,834.9 | 569,729.5 | 594,760.9 | 94.1 | 735.0 | 73.7 | 12.5 | 108.8 | 11.0 | 7.5 | 0.3 | 0.5 | 0.0 | 0.9 | 3.7 | 1.0 | 0.2 | 1.2 | 6.8 |
|  | **Shared domestic tap** | 123 | 303.0 | 704,812.8 | 2,087,119.4 | 3,756,643.0 | 521,387.5 | 593,087.4 | 69.3 | 753.2 | 72.8 | 14.6 | 96.3 | 8.8 | 6.9 | 0.3 | 0.5 | 0.0 | 1.2 | 5.1 | 1.1 | 0.2 | 1.1 | 6.1 |
|  | **Communal Water kiosk** | 105 | 192.8 | 615,129.6 | 2,147,504.0 | 2,383,744.8 | 510,944.9 | 471,363.8 | 62.5 | 519.4 | 72.9 | 9.4 | 69.7 | 9.1 | 7.6 | 0.3 | 0.5 | 0.0 | 0.9 | 3.8 | 0.7 | 0.3 | 0.9 | 5.4 |
|  | **Protected well** | 34 | 307.3 | 699,638.2 | 2,457,045.5 | 6,653,640.1 | 570,275.8 | 610,338.1 | 91.5 | 792.8 | 81.6 | 17.1 | 118.4 | 10.7 | 11.0 | 0.3 | 0.6 | 0.0 | 1.3 | 6.2 | 1.2 | 0.2 | 1.0 | 6.5 |
|  | **Lake/unprotected well** | 12 | 364.2 | 896,407.5 | 1,973,017.7 | 2,091,800.6 | 656,199.0 | 610,707.3 | 90.4 | 815.3 | 86.0 | 38.4 | 128.8 | 17.9 | 8.0 | 0.1 | 0.6 | 0.0 | 1.1 | 9.8 | 1.7 | 0.3 | 0.9 | 8.3 |
| **Fridge** | **No** | 262 | 283.2 | 592,200.1 | 2,008,983.4 | 3,125,172.1 | 547,210.9 | 498,130.0 | 71.4 | 461.6 | 70.5 | 8.3 | 85.1 | 7.7 | 8.2 | 0.4 | 0.5 | 0.0 | 1.1 | 4.7 | 1.0 | 0.2 | 1.3 | 5.6 |
|  | **Yes** | 106 | 277.4 | 641,515.4 | 2,623,787.1 | 4,405,932.1 | 513,339.6 | 578,707.4 | 67.2 | 673.2 | 83.4 | 14.4 | 84.5 | 8.7 | 8.1 | 0.3 | 0.5 | 0.0 | 1.2 | 4.3 | 0.9 | 0.2 | 1.1 | 6.0 |
| **TV** | **No** | 208 | 251.8 | 568,959.5 | 1,804,590.8 | 2,477,268.4 | 537,676.8 | 476,697.2 | 64.6 | 410.3 | 69.4 | 8.1 | 77.9 | 6.2 | 7.9 | 0.3 | 0.5 | 0.0 | 1.1 | 4.3 | 0.8 | 0.2 | 1.4 | 5.3 |
|  | **Yes** | 160 | 288.4 | 707,910.5 | 2,980,046.6 | 5,008,326.5 | 511,609.6 | 579,480.1 | 71.8 | 775.1 | 78.9 | 16.6 | 105.7 | 10.8 | 9.7 | 0.3 | 0.5 | 0.0 | 1.2 | 5.4 | 1.0 | 0.2 | 0.9 | 6.6 |
| **Video** | **No** | 243 | 275.4 | 577,916.7 | 1,973,017.7 | 3,410,258.9 | 547,286.1 | 530,968.0 | 72.7 | 513.4 | 74.9 | 8.9 | 86.3 | 7.0 | 8.5 | 0.3 | 0.5 | 0.0 | 1.1 | 4.3 | 0.9 | 0.2 | 1.3 | 5.6 |
|  | **Yes** | 125 | 284.3 | 689,659.7 | 2,080,534.1 | 4,270,520.5 | 496,273.8 | 547,756.6 | 69.9 | 672.4 | 74.3 | 17.1 | 95.3 | 10.5 | 7.8 | 0.3 | 0.5 | 0.0 | 1.2 | 5.4 | 1.0 | 0.2 | 1.0 | 6.4 |
| **Radio** | **No** | 126 | 304.4 | 665,366.6 | 2,897,073.6 | 5,163,789.7 | 557,121.0 | 537,921.5 | 72.6 | 562.1 | 70.5 | 9.2 | 95.1 | 9.9 | 10.2 | 0.5 | 0.5 | 0.0 | 1.5 | 6.2 | 1.3 | 0.2 | 1.3 | 6.0 |
|  | **Yes** | 242 | 272.9 | 674,207.8 | 2,046,443.5 | 3,695,345.6 | 521,387.5 | 573,805.8 | 71.4 | 658.3 | 76.8 | 13.0 | 87.0 | 9.2 | 8.1 | 0.2 | 0.5 | 0.0 | 1.0 | 4.5 | 0.9 | 0.2 | 1.0 | 6.0 |
| **Mobile phone** | **No** | 78 | 309.8 | 770,271.9 | 2,093,704.7 | 5,575,708.8 | 596,307.2 | 596,405.6 | 84.2 | 526.3 | 70.6 | 10.2 | 101.1 | 10.3 | 8.8 | 0.2 | 0.5 | 0.0 | 1.7 | 6.2 | 1.2 | 0.2 | 1.2 | 5.6 |
|  | **Yes** | 290 | 281.8 | 688,292.8 | 2,457,045.5 | 4,270,520.5 | 524,512.2 | 581,866.6 | 71.8 | 705.2 | 76.0 | 14.7 | 91.2 | 9.5 | 8.5 | 0.3 | 0.5 | 0.0 | 1.2 | 4.7 | 1.0 | 0.2 | 1.0 | 6.4 |
| **Bicycle** | **No** | 322 | 285.4 | 570,494.2 | 2,008,983.4 | 3,695,345.6 | 523,537.1 | 530,968.0 | 71.4 | 484.3 | 70.5 | 8.7 | 87.5 | 7.7 | 8.2 | 0.3 | 0.5 | 0.0 | 1.1 | 5.2 | 1.0 | 0.2 | 1.1 | 5.3 |
|  | **Yes** | 46 | 197.9 | 578,163.5 | 4,248,403.0 | 3,963,184.5 | 514,141.2 | 586,734.1 | 57.0 | 496.7 | 73.6 | 12.3 | 70.1 | 6.5 | 6.3 | 0.1 | 0.4 | 0.0 | 1.1 | 2.5 | 0.7 | 0.3 | 0.9 | 5.1 |
| **Car** | **No** | 352 | 273.4 | 570,275.5 | 1,973,017.7 | 3,614,555.3 | 520,117.6 | 488,727.9 | 64.8 | 465.9 | 70.5 | 8.4 | 84.6 | 7.0 | 8.1 | 0.3 | 0.5 | 0.0 | 1.1 | 4.4 | 0.9 | 0.2 | 1.2 | 5.2 |
|  | **Yes** | 16 | 366.5 | 589,326.6 | 4,332,352.7 | 10,061,112.7 | 470,538.4 | 599,810.0 | 110.9 | 613.8 | 96.2 | 16.2 | 120.2 | 11.9 | 4.9 | 0.1 | 0.5 | 0.0 | 1.2 | 4.9 | 1.1 | 0.2 | 0.5 | 3.6 |
| **Number of adults** | **1** | 57 | 286.9 | 828,244.5 | 2,370,497.5 | 3,408,991.3 | 557,195.5 | 574,366.4 | 75.2 | 787.1 | 75.3 | 7.3 | 87.8 | 8.0 | 11.0 | 0.5 | 0.6 | 0.0 | 1.2 | 3.6 | 1.3 | 0.2 | 1.0 | 6.7 |
|  | **2** | 182 | 266.9 | 666,673.9 | 2,008,983.4 | 3,594,806.8 | 531,723.4 | 584,607.8 | 73.1 | 584.8 | 72.8 | 15.8 | 87.2 | 10.5 | 7.5 | 0.3 | 0.5 | 0.0 | 1.1 | 4.9 | 1.0 | 0.2 | 1.0 | 6.0 |
|  | **3** | 69 | 341.5 | 775,529.9 | 4,079,811.1 | 11,309,793.6 | 547,772.6 | 612,919.1 | 77.8 | 932.1 | 79.0 | 19.5 | 134.3 | 11.0 | 13.1 | 0.4 | 0.5 | 0.0 | 1.4 | 6.7 | 1.0 | 0.2 | 1.1 | 6.6 |
|  | **>3** | 59 | 293.0 | 643,865.6 | 1,605,520.7 | 5,087,397.9 | 548,175.4 | 562,555.5 | 71.8 | 767.4 | 86.3 | 14.4 | 95.8 | 8.8 | 8.4 | 0.1 | 0.5 | 0.0 | 1.3 | 4.4 | 1.4 | 0.2 | 1.1 | 6.9 |
| **Number of children** | **0** | 64 | 287.4 | 656,691.6 | 2,810,115.9 | 11,101,747.3 | 560,328.3 | 534,944.0 | 69.4 | 572.4 | 75.3 | 10.4 | 91.6 | 6.7 | 12.8 | 0.3 | 0.6 | 0.0 | 1.5 | 4.3 | 1.3 | 0.2 | 1.1 | 7.1 |
|  | **1** | 65 | 292.6 | 876,094.0 | 1,607,269.9 | 3,125,172.1 | 551,483.5 | 596,150.9 | 78.7 | 772.1 | 78.0 | 23.2 | 99.0 | 10.5 | 6.6 | 0.4 | 0.5 | 0.0 | 1.0 | 5.9 | 1.0 | 0.2 | 1.0 | 5.2 |
|  | **2** | 96 | 250.7 | 699,638.2 | 2,260,691.6 | 4,007,018.9 | 518,847.7 | 593,638.2 | 74.4 | 860.2 | 76.8 | 20.0 | 98.9 | 10.6 | 11.7 | 0.2 | 0.6 | 0.0 | 1.2 | 4.8 | 1.2 | 0.2 | 1.1 | 6.8 |
|  | **3** | 79 | 342.6 | 754,544.8 | 2,210,860.2 | 2,992,650.0 | 560,401.4 | 561,917.3 | 84.4 | 748.1 | 78.7 | 13.3 | 102.6 | 12.4 | 8.4 | 0.4 | 0.5 | 0.0 | 1.2 | 6.4 | 1.2 | 0.2 | 1.0 | 7.1 |
|  | **>3** | 69 | 260.1 | 597,252.9 | 3,070,050.5 | 5,241,060.4 | 490,414.1 | 545,441.1 | 70.3 | 524.0 | 73.4 | 11.7 | 84.6 | 7.8 | 7.9 | 0.1 | 0.5 | 0.0 | 1.2 | 3.6 | 1.0 | 0.2 | 0.9 | 5.3 |
| **Education** | **None** | 27 | 284.9 | 814,557.7 | 4,075,258.8 | 4,906,924.4 | 578,109.9 | 663,435.7 | 83.9 | 1,177.4 | 74.6 | 39.7 | 148.7 | 20.9 | 13.7 | 0.2 | 0.5 | 0.0 | 1.8 | 10.1 | 0.8 | 0.2 | 1.0 | 8.5 |
|  | **Primary incomplete** | 116 | 292.6 | 779,496.9 | 2,355,399.5 | 4,382,122.7 | 535,292.8 | 587,289.7 | 81.9 | 797.4 | 86.3 | 11.1 | 87.0 | 8.0 | 9.6 | 0.3 | 0.6 | 0.0 | 1.1 | 6.0 | 1.3 | 0.2 | 1.1 | 6.5 |
|  | **Primary complete** | 43 | 281.8 | 777,094.5 | 3,352,892.6 | 4,388,178.2 | 589,650.7 | 642,828.6 | 89.7 | 580.7 | 71.5 | 32.8 | 131.8 | 10.4 | 10.7 | 0.4 | 0.5 | 0.0 | 1.4 | 6.4 | 1.0 | 0.2 | 0.9 | 7.2 |
|  | **Secondary incomplete** | 96 | 296.8 | 602,863.5 | 1,582,304.2 | 4,357,181.9 | 545,061.5 | 567,457.1 | 71.3 | 521.1 | 75.9 | 11.5 | 88.5 | 9.9 | 9.9 | 0.2 | 0.5 | 0.0 | 1.1 | 4.2 | 1.1 | 0.2 | 1.1 | 5.9 |
|  | **Secondary complete** | 67 | 249.5 | 671,701.7 | 2,630,961.0 | 4,050,644.4 | 492,901.6 | 560,850.1 | 70.5 | 784.5 | 74.8 | 15.9 | 95.1 | 10.6 | 7.8 | 0.4 | 0.5 | 0.0 | 1.2 | 4.4 | 0.9 | 0.2 | 0.9 | 5.8 |
|  | **Tertiary complete** | 26 | 270.3 | 693,980.1 | 1,093,508.6 | 2,758,471.5 | 517,752.1 | 532,977.5 | 61.1 | 487.2 | 69.5 | 8.4 | 84.8 | 7.3 | 4.1 | 0.1 | 0.2 | 0.0 | 1.0 | 3.5 | 1.0 | 0.2 | 1.1 | 5.2 |
| **Occupation** | **Unemployed** | 75 | 321.4 | 784,640.1 | 2,684,191.3 | 6,692,766.4 | 541,029.6 | 607,717.3 | 81.7 | 868.4 | 77.8 | 23.2 | 141.8 | 10.2 | 12.4 | 0.4 | 0.6 | 0.0 | 1.5 | 5.3 | 1.3 | 0.2 | 1.2 | 7.7 |
|  | **Student** | 55 | 278.4 | 533,739.9 | 1,242,662.4 | 2,169,746.2 | 520,117.6 | 529,633.8 | 62.4 | 575.5 | 70.0 | 9.5 | 90.0 | 9.0 | 4.6 | 0.1 | 0.4 | 0.0 | 1.1 | 4.4 | 1.3 | 0.1 | 1.0 | 5.3 |
|  | **Non-skilled labourer** | 197 | 279.3 | 695,564.1 | 2,079,878.4 | 3,838,205.0 | 552,702.0 | 593,362.8 | 76.5 | 702.1 | 76.8 | 15.3 | 87.0 | 10.7 | 9.3 | 0.3 | 0.5 | 0.0 | 1.2 | 5.6 | 1.0 | 0.2 | 1.0 | 6.5 |
|  | **Skilled labourer** | 32 | 224.0 | 653,576.6 | 4,959,358.5 | 3,798,089.9 | 476,911.8 | 534,944.0 | 77.0 | 516.9 | 78.3 | 8.4 | 84.8 | 7.7 | 8.4 | 0.2 | 0.6 | 0.0 | 0.9 | 3.1 | 1.0 | 0.2 | 1.5 | 5.9 |
|  | **Professional worker** | 15 | 305.7 | 754,544.8 | 2,630,961.0 | 7,433,241.3 | 426,103.8 | 480,180.1 | 53.7 | 459.5 | 63.7 | 13.7 | 105.7 | 8.6 | 5.3 | 1.6 | 0.4 | 0.0 | 0.7 | 4.5 | 0.9 | 0.1 | 0.8 | 5.4 |
| **Employment status** | **Full time** | 191 | 244.9 | 679,313.8 | 1,632,553.9 | 2,241,036.2 | 518,847.7 | 547,467.9 | 70.0 | 511.5 | 73.9 | 12.7 | 77.7 | 8.8 | 6.3 | 0.3 | 0.5 | 0.0 | 0.9 | 4.4 | 0.8 | 0.2 | 0.9 | 5.3 |
|  | **Part time** | 25 | 320.4 | 770,271.9 | 6,500,476.0 | 13,649,747.8 | 617,591.8 | 660,716.8 | 76.6 | 870.7 | 73.6 | 19.0 | 109.1 | 11.6 | 13.3 | 0.3 | 0.6 | 0.1 | 1.5 | 7.8 | 1.4 | 0.2 | 1.5 | 6.6 |
|  | **Not working - ill health** | 12 | 229.8 | 649,444.8 | 3,086,409.5 | 3,220,584.4 | 538,618.5 | 613,683.3 | 55.0 | 886.2 | 91.4 | 46.1 | 77.7 | 7.4 | 11.0 | 0.1 | 0.7 | 0.1 | 1.9 | 3.8 | 1.1 | 0.2 | 1.0 | 8.0 |
|  | **Not working - lack of employment** | 44 | 292.6 | 843,143.3 | 1,616,616.5 | 4,486,264.7 | 490,414.1 | 577,540.6 | 68.5 | 858.4 | 73.9 | 27.2 | 90.9 | 12.2 | 11.7 | 0.3 | 0.6 | 0.0 | 1.0 | 4.6 | 1.0 | 0.3 | 1.1 | 6.9 |
| **Working days per week** | **4 or less** | 13 | 303.7 | 641,808.7 | 20,277,683.7 | 19,686,072.5 | 528,850.8 | 608,622.4 | 62.2 | 544.9 | 69.7 | 16.5 | 100.1 | 13.1 | 9.3 | 0.3 | 0.5 | 0.0 | 1.4 | 5.7 | 0.9 | 0.2 | 1.6 | 6.1 |
|  | **5** | 49 | 252.2 | 503,533.4 | 1,487,635.9 | 1,819,679.3 | 427,785.5 | 450,070.9 | 55.1 | 327.8 | 62.8 | 5.1 | 60.4 | 5.2 | 3.8 | 0.2 | 0.3 | 0.0 | 0.8 | 2.5 | 0.6 | 0.2 | 0.8 | 3.5 |
|  | **6** | 71 | 189.5 | 753,021.3 | 1,500,817.1 | 2,858,008.7 | 544,663.9 | 581,710.1 | 61.5 | 562.1 | 65.5 | 12.5 | 84.8 | 8.4 | 6.1 | 0.3 | 0.5 | 0.0 | 0.8 | 3.8 | 0.8 | 0.2 | 0.6 | 6.1 |
|  | **7** | 37 | 274.3 | 607,744.2 | 1,890,450.7 | 4,177,772.9 | 522,150.0 | 579,652.7 | 76.5 | 701.3 | 89.0 | 16.2 | 110.0 | 9.8 | 7.7 | 0.3 | 0.5 | 0.0 | 1.2 | 6.1 | 0.9 | 0.2 | 0.9 | 6.5 |
| **Working hours per day** | **Less than 8** | 32 | 281.7 | 789,099.0 | 2,182,709.4 | 3,820,195.8 | 451,507.8 | 509,393.0 | 75.5 | 506.2 | 71.3 | 14.8 | 86.7 | 8.0 | 7.6 | 0.2 | 0.6 | 0.0 | 1.1 | 3.1 | 0.9 | 0.2 | 1.0 | 5.1 |
|  | **8** | 81 | 181.3 | 556,012.8 | 970,376.0 | 1,527,927.8 | 449,647.8 | 457,860.0 | 55.9 | 435.4 | 65.4 | 6.6 | 62.8 | 5.9 | 4.3 | 0.2 | 0.3 | 0.0 | 0.6 | 2.6 | 0.6 | 0.2 | 0.9 | 3.5 |
|  | **9 to 12** | 70 | 281.8 | 885,109.5 | 2,080,534.1 | 5,806,829.8 | 562,816.3 | 626,882.7 | 84.2 | 784.5 | 78.0 | 14.9 | 92.4 | 11.5 | 10.2 | 0.3 | 0.5 | 0.0 | 1.1 | 6.6 | 1.0 | 0.2 | 0.7 | 7.1 |
|  | **More than 12** | 12 | 273.0 | 502,853.7 | 3,775,008.0 | 4,529,485.5 | 488,683.9 | 589,863.5 | 48.8 | 525.2 | 76.1 | 16.7 | 81.1 | 11.6 | 6.6 | 0.1 | 0.5 | 0.0 | 1.6 | 4.5 | 1.0 | 0.2 | 0.6 | 7.1 |
| **Paid by Salary** | **No** | 235 | 314.0 | 674,501.7 | 5,203,573.0 | 7,088,253.0 | 560,256.9 | 587,397.7 | 76.4 | 792.8 | 77.2 | 14.5 | 109.1 | 8.4 | 11.2 | 0.3 | 0.6 | 0.0 | 1.2 | 5.5 | 1.5 | 0.2 | 0.9 | 6.4 |
|  | **Yes** | 133 | 234.2 | 641,584.6 | 1,252,894.9 | 2,177,309.2 | 516,464.7 | 531,079.2 | 61.3 | 503.9 | 71.7 | 13.0 | 76.9 | 9.6 | 6.4 | 0.3 | 0.4 | 0.0 | 0.8 | 4.7 | 0.8 | 0.2 | 0.8 | 5.4 |
| **Participant income (USD/month)** | **<10** | 64 | 266.9 | 860,968.2 | 2,267,527.5 | 3,919,767.0 | 562,816.3 | 587,505.6 | 86.6 | 893.6 | 94.2 | 16.6 | 70.4 | 11.0 | 8.0 | 0.3 | 0.6 | 0.0 | 1.2 | 6.4 | 1.0 | 0.2 | 0.9 | 7.1 |
|  | **Oct-25** | 44 | 313.0 | 567,031.7 | 3,261,736.1 | 3,521,118.0 | 544,953.8 | 581,137.9 | 73.4 | 759.8 | 80.1 | 13.9 | 117.2 | 11.8 | 9.7 | 0.5 | 0.6 | 0.0 | 0.9 | 6.1 | 1.4 | 0.2 | 0.8 | 7.0 |
|  | **26 - 50** | 65 | 249.7 | 616,154.3 | 1,607,269.9 | 2,086,227.0 | 545,852.3 | 530,968.0 | 62.9 | 410.3 | 61.8 | 8.1 | 62.9 | 5.6 | 5.8 | 1.0 | 0.4 | 0.0 | 1.0 | 4.3 | 0.8 | 0.2 | 1.4 | 5.1 |
|  | **51 - 100** | 46 | 286.3 | 570,056.8 | 1,734,457.8 | 3,533,765.1 | 527,583.9 | 548,938.5 | 71.4 | 562.1 | 79.6 | 11.8 | 95.2 | 6.9 | 5.7 | 0.2 | 0.5 | 0.0 | 1.0 | 5.5 | 0.8 | 0.2 | 1.0 | 4.8 |
|  | **>100** | 36 | 282.4 | 754,544.8 | 11,912,554.5 | 11,177,038.7 | 431,952.7 | 545,441.1 | 53.7 | 487.2 | 69.4 | 10.2 | 91.2 | 7.3 | 10.3 | 0.3 | 0.5 | 0.0 | 1.2 | 2.8 | 1.2 | 0.2 | 1.0 | 5.2 |
| **Household income (USD / month)** | **<10** | 62 | 264.1 | 859,506.5 | 2,241,656.4 | 3,985,205.7 | 559,984.8 | 587,397.7 | 78.7 | 865.5 | 89.6 | 16.3 | 69.0 | 11.3 | 8.1 | 0.3 | 0.6 | 0.0 | 1.2 | 5.6 | 1.0 | 0.2 | 0.8 | 7.0 |
|  | **Oct-25** | 29 | 351.8 | 752,005.7 | 4,379,326.1 | 6,431,777.6 | 574,621.0 | 590,935.9 | 69.6 | 796.4 | 75.0 | 15.3 | 184.0 | 11.7 | 7.8 | 0.4 | 0.6 | 0.0 | 1.0 | 6.7 | 1.6 | 0.2 | 0.8 | 6.6 |
|  | **26 - 50** | 57 | 267.6 | 581,347.2 | 1,165,964.1 | 1,449,260.9 | 553,474.3 | 574,807.7 | 71.7 | 409.3 | 63.3 | 9.5 | 68.6 | 5.8 | 5.9 | 0.4 | 0.4 | 0.0 | 1.2 | 4.3 | 0.7 | 0.2 | 1.4 | 5.2 |
|  | **51 - 100** | 49 | 286.3 | 570,056.8 | 2,355,399.5 | 4,010,375.1 | 547,286.1 | 544,281.3 | 68.4 | 674.0 | 81.6 | 9.2 | 77.9 | 7.1 | 12.4 | 0.3 | 0.5 | 0.0 | 1.1 | 3.9 | 1.5 | 0.2 | 1.2 | 5.6 |
|  | **>100** | 54 | 262.5 | 674,501.7 | 10,492,944.2 | 8,387,153.3 | 423,746.0 | 524,144.0 | 51.5 | 454.5 | 65.5 | 10.8 | 88.0 | 6.9 | 6.0 | 0.3 | 0.5 | 0.0 | 1.2 | 5.3 | 0.8 | 0.2 | 1.0 | 5.0 |
| **In receipt of benefits** | **No** | 341 | 274.4 | 607,683.5 | 2,008,983.4 | 4,007,018.9 | 531,723.4 | 546,990.0 | 68.4 | 522.3 | 73.8 | 12.5 | 87.5 | 8.6 | 8.2 | 0.2 | 0.5 | 0.0 | 1.1 | 4.4 | 0.9 | 0.2 | 1.1 | 5.7 |
|  | **Yes** | 27 | 294.3 | 835,238.7 | 3,273,664.0 | 5,597,268.0 | 523,437.5 | 532,998.5 | 69.8 | 837.4 | 97.4 | 24.5 | 158.8 | 10.6 | 7.3 | 0.5 | 0.5 | 0.0 | 1.3 | 6.6 | 1.3 | 0.2 | 1.0 | 7.0 |
| **In receipt of household credit** | **No** |  | 299.2 | 800,487.2 | 2,505,720.8 | 5,535,528.5 | 557,237.6 | 602,061.5 | 83.5 | 752.9 | 77.8 | 15.8 | 99.0 | 11.0 | 10.7 | 0.3 | 0.6 | 0.0 | 1.4 | 5.9 | 1.0 | 0.2 | 1.0 | 6.8 |
|  | **Yes** | 169 | 255.2 | 598,043.5 | 2,091,550.6 | 3,594,806.8 | 512,562.5 | 502,846.8 | 69.6 | 537.9 | 72.7 | 14.5 | 85.4 | 8.2 | 7.6 | 0.3 | 0.5 | 0.0 | 1.1 | 3.9 | 1.1 | 0.2 | 1.0 | 5.3 |
| **Source of credit** | **Family** | 20 | 324.0 | 769,408.3 | 1,605,520.7 | 6,034,023.9 | 562,816.3 | 646,232.3 | 113.2 | 1,040.5 | 73.7 | 16.7 | 145.1 | 21.9 | 12.9 | 0.4 | 0.5 | 0.0 | 1.2 | 7.1 | 1.2 | 0.3 | 1.0 | 6.7 |
|  | **Friends** | 73 | 212.0 | 593,164.4 | 1,466,292.5 | 2,207,656.3 | 524,586.9 | 482,111.4 | 69.9 | 459.7 | 68.5 | 12.9 | 88.8 | 6.9 | 5.1 | 0.2 | 0.4 | 0.0 | 0.9 | 3.6 | 0.8 | 0.2 | 1.0 | 5.3 |
|  | **Bank** | 24 | 264.1 | 639,303.6 | 3,163,186.2 | 4,466,693.5 | 477,576.0 | 601,241.8 | 60.0 | 829.5 | 83.6 | 11.3 | 91.2 | 9.2 | 10.3 | 0.2 | 0.4 | 0.0 | 1.2 | 2.9 | 1.3 | 0.3 | 0.7 | 5.3 |
|  | **Co-operative** | 48 | 257.4 | 497,291.1 | 3,454,192.1 | 3,594,806.8 | 405,110.4 | 446,089.1 | 56.8 | 361.1 | 73.0 | 8.3 | 74.4 | 6.6 | 6.9 | 0.4 | 0.5 | 0.0 | 1.1 | 4.5 | 1.2 | 0.2 | 1.2 | 4.4 |
| **Household has enough food** | **No** | 339 | 286.3 | 691,490.0 | 2,365,338.2 | 4,345,170.6 | 538,957.2 | 587,289.7 | 72.8 | 753.2 | 75.9 | 14.6 | 92.4 | 9.9 | 9.6 | 0.3 | 0.5 | 0.0 | 1.2 | 5.2 | 1.1 | 0.2 | 1.0 | 6.5 |
|  | **Yes** | 29 | 222.1 | 686,925.9 | 1,263,436.3 | 2,110,039.6 | 576,523.0 | 572,946.7 | 77.0 | 306.5 | 62.9 | 12.8 | 87.5 | 11.8 | 3.3 | 0.6 | 0.5 | 0.0 | 0.6 | 4.3 | 0.6 | 0.2 | 1.2 | 5.2 |
| **Household grows crops** | **No** | 305 | 284.5 | 769,840.1 | 2,355,399.5 | 4,010,375.1 | 544,663.9 | 587,816.6 | 71.9 | 752.9 | 75.9 | 15.8 | 95.2 | 10.0 | 9.6 | 0.3 | 0.5 | 0.0 | 1.2 | 5.0 | 1.0 | 0.2 | 1.0 | 6.5 |
|  | **Yes** | 63 | 281.8 | 564,016.8 | 2,116,854.6 | 4,143,970.7 | 534,261.6 | 561,509.4 | 91.5 | 524.1 | 70.2 | 11.2 | 77.3 | 10.3 | 8.2 | 0.3 | 0.5 | 0.0 | 1.1 | 6.9 | 1.0 | 0.2 | 1.0 | 5.7 |
| **Households with a child that has left school due to lack of food** | **No** | 359 | 283.4 | 689,747.6 | 2,267,527.5 | 4,307,845.6 | 545,459.1 | 581,154.1 | 72.7 | 707.4 | 75.5 | 14.0 | 91.6 | 10.2 | 8.9 | 0.3 | 0.5 | 0.0 | 1.2 | 5.2 | 1.0 | 0.2 | 1.0 | 6.4 |
|  | **Yes** | 9 | 294.2 | 600,445.6 | 1,340,591.1 | 2,845,925.4 | 529,563.5 | 631,108.1 | 60.2 | 434.6 | 66.5 | 31.2 | 151.8 | 7.8 | 6.6 | 0.5 | 0.5 | 0.0 | 0.8 | 3.0 | 0.8 | 0.2 | 0.9 | 6.3 |
| **Mode of transport** | **Walking** | 48 | 264.1 | 570,056.8 | 1,904,982.6 | 2,477,268.4 | 518,812.7 | 534,364.7 | 84.2 | 674.0 | 74.8 | 12.8 | 59.4 | 11.0 | 8.4 | 0.1 | 0.5 | 0.0 | 1.0 | 4.8 | 0.9 | 0.2 | 0.8 | 6.4 |
|  | **Bus** | 302 | 2,868.3 | 1,033,431.4 | 18,572,429.1 | 110,984,621.4 | 839,631.5 | 1,468,353.1 | 89.0 | 1,150,430.1 | 118.8 | 23.6 | 324.5 | 15.5 | 161.5 | 4.8 | 0.8 | 0.0 | 19.9 | 15.1 | 1.9 | 0.3 | 0.9 | 14.9 |
|  | **Car** | 8 | 294.5 | 730,028.8 | 2,355,399.5 | 4,160,582.5 | 545,852.3 | 581,866.6 | 71.8 | 719.8 | 75.0 | 14.7 | 95.1 | 9.9 | 8.9 | 0.3 | 0.5 | 0.0 | 1.2 | 4.7 | 1.1 | 0.2 | 1.0 | 6.4 |
|  | **Other** | 3 | 353.5 | 754,277.3 | 4,332,352.7 | 4,697,679.0 | 518,452.9 | 630,568.7 | 95.1 | 1,773.5 | 109.8 | 40.7 | 84.3 | 19.2 | 7.6 | 0.2 | 0.3 | 0.0 | 2.7 | 8.6 | 1.8 | 0.2 | 0.6 | 5.0 |
| **Transport costs (Malawian Kwacha)** | **200 - 350** | 134 | 299.3 | 834,057.3 | 2,475,859.4 | 4,757,081.2 | 553,920.6 | 600,191.9 | 71.2 | 793.7 | 73.5 | 21.9 | 102.3 | 10.7 | 8.0 | 0.3 | 0.5 | 0.0 | 1.1 | 4.5 | 1.0 | 0.2 | 1.0 | 6.8 |
|  | **350 - 600** | 64 | 243.4 | 559,089.7 | 968,995.5 | 2,440,084.9 | 498,363.4 | 481,307.5 | 60.9 | 440.7 | 78.1 | 8.2 | 81.4 | 7.2 | 8.0 | 0.2 | 0.5 | 0.0 | 1.0 | 4.0 | 0.8 | 0.2 | 1.0 | 5.0 |
|  | **600 - 1000** | 45 | 307.4 | 541,057.5 | 7,128,376.6 | 4,725,093.7 | 540,394.0 | 614,820.8 | 72.6 | 592.1 | 77.8 | 13.0 | 72.0 | 5.7 | 12.5 | 0.4 | 0.6 | 0.0 | 1.2 | 7.8 | 1.2 | 0.3 | 1.5 | 5.9 |
|  | **>1000** | 61 | 359.5 | 721,648.1 | 3,082,548.0 | 5,276,260.8 | 592,126.6 | 590,296.5 | 91.5 | 730.3 | 75.4 | 9.8 | 110.1 | 10.1 | 8.5 | 0.3 | 0.5 | 0.0 | 1.5 | 5.9 | 1.3 | 0.2 | 1.0 | 6.2 |
| **Travel time** | **<10** | 8 | 213.0 | 718,153.9 | 1,288,615.0 | 3,934,578.1 | 503,553.3 | 512,221.1 | 63.1 | 596.5 | 80.2 | 20.5 | 138.6 | 9.4 | 5.9 | 0.0 | 0.5 | 0.0 | 1.0 | 3.3 | 1.5 | 0.2 | 0.7 | 6.9 |
|  | **Oct-30** | 38 | 261.1 | 533,739.9 | 1,904,982.6 | 3,237,473.9 | 460,403.4 | 550,072.1 | 78.3 | 490.8 | 65.4 | 14.7 | 71.4 | 10.6 | 4.0 | 0.6 | 0.4 | 0.0 | 0.9 | 5.9 | 0.9 | 0.2 | 1.1 | 5.1 |
|  | **31 - 60** | 136 | 307.4 | 653,576.6 | 3,216,917.9 | 5,234,399.0 | 535,292.8 | 556,206.8 | 69.9 | 772.1 | 79.7 | 12.9 | 76.7 | 8.9 | 8.6 | 0.3 | 0.6 | 0.0 | 1.2 | 5.1 | 1.1 | 0.2 | 1.0 | 6.4 |
|  | **61 - 120** | 140 | 286.3 | 769,408.3 | 1,721,665.9 | 3,594,806.8 | 557,936.9 | 600,535.0 | 78.7 | 646.9 | 75.0 | 14.0 | 106.7 | 10.9 | 10.6 | 0.3 | 0.5 | 0.0 | 1.3 | 5.7 | 1.1 | 0.2 | 1.1 | 6.4 |
|  | **>120** | 23 | 241.5 | 622,620.6 | 3,545,541.7 | 4,442,884.2 | 506,235.6 | 556,798.3 | 70.2 | 765.6 | 74.3 | 11.5 | 86.2 | 7.5 | 9.4 | 0.1 | 0.6 | 0.0 | 1.0 | 3.6 | 0.8 | 0.2 | 1.0 | 6.7 |
| **Health visual analogue score** | **0** | 108 | 270.3 | 809,834.9 | 3,325,789.7 | 4,866,710.1 | 550,274.1 | 597,440.0 | 85.2 | 865.4 | 84.6 | 20.0 | 88.2 | 10.8 | 8.2 | 0.3 | 0.6 | 0.0 | 1.1 | 5.3 | 1.2 | 0.2 | 0.9 | 6.6 |
|  | **1** | 128 | 281.8 | 627,729.0 | 1,607,269.9 | 2,793,415.3 | 528,605.4 | 546,990.0 | 63.5 | 526.1 | 70.6 | 9.8 | 84.9 | 7.8 | 8.2 | 0.1 | 0.5 | 0.0 | 0.9 | 3.8 | 0.9 | 0.2 | 1.0 | 5.8 |
|  | **2** | 85 | 327.7 | 719,308.1 | 2,093,704.7 | 5,615,889.2 | 542,499.0 | 602,218.7 | 73.9 | 787.2 | 74.6 | 23.7 | 118.4 | 12.4 | 11.0 | 0.5 | 0.6 | 0.0 | 1.6 | 5.9 | 1.1 | 0.2 | 1.1 | 7.5 |
|  | **3** | 28 | 264.5 | 739,567.6 | 2,148,159.7 | 3,995,935.1 | 514,637.0 | 600,191.9 | 71.9 | 757.6 | 77.5 | 16.3 | 89.7 | 9.2 | 16.1 | 0.8 | 0.5 | 0.0 | 1.2 | 4.2 | 1.1 | 0.2 | 1.0 | 6.7 |
|  | **4** | 12 | 294.8 | 614,769.7 | 2,877,120.9 | 6,150,055.2 | 630,636.5 | 532,956.0 | 69.6 | 847.1 | 79.6 | 27.1 | 177.0 | 9.9 | 12.1 | 0.3 | 0.5 | 0.0 | 0.8 | 13.0 | 1.1 | 0.4 | 0.9 | 8.1 |
|  | **5** | 3 | 500.5 | 397,186.8 | 3,216,917.9 | 2,106,709.8 | 335,316.7 | 578,707.4 | 41.7 | 522.3 | 109.1 | 14.5 | 77.3 | 10.5 | 3.7 | 0.4 | 0.2 | 0.0 | 11.1 | 18.8 | 1.1 | 0.2 | 2.1 | 2.8 |
| **Wellbeing visual analogue score** | **0** | 107 | 269.7 | 734,333.4 | 3,325,789.7 | 3,840,919.3 | 550,274.1 | 597,440.0 | 73.0 | 845.6 | 80.8 | 15.8 | 80.3 | 9.7 | 8.6 | 0.2 | 0.6 | 0.0 | 1.1 | 4.5 | 1.2 | 0.2 | 1.0 | 6.5 |
|  | **1** | 140 | 281.1 | 734,523.3 | 1,943,341.1 | 2,608,948.9 | 521,192.4 | 546,990.0 | 70.3 | 580.7 | 71.6 | 12.1 | 88.0 | 9.2 | 9.9 | 0.2 | 0.5 | 0.0 | 1.2 | 4.1 | 1.0 | 0.2 | 1.0 | 5.9 |
|  | **2** | 82 | 339.4 | 654,153.0 | 2,093,704.7 | 5,615,889.2 | 558,720.4 | 562,391.7 | 69.3 | 730.7 | 74.8 | 9.4 | 92.4 | 9.3 | 8.0 | 0.6 | 0.5 | 0.0 | 1.3 | 4.7 | 1.0 | 0.2 | 1.0 | 6.6 |
|  | **3** | 23 | 432.4 | 628,783.3 | 2,026,775.9 | 5,499,683.5 | 555,344.6 | 721,244.0 | 118.2 | 819.0 | 79.4 | 45.9 | 240.5 | 12.0 | 11.7 | 0.3 | 0.5 | 0.1 | 1.5 | 9.1 | 1.0 | 0.3 | 1.0 | 7.5 |
|  | **4** | 9 | 390.2 | 568,181.3 | 3,143,484.2 | 10,014,036.3 | 495,428.5 | 553,554.1 | 70.8 | 675.3 | 88.7 | 35.0 | 191.2 | 9.8 | 5.4 | 0.4 | 0.4 | 0.0 | 1.2 | 8.7 | 2.7 | 0.2 | 0.8 | 6.1 |
|  | **5** | 2 | 388.0 | 724,716.3 | 126,332,759.1 | 81,847,914.0 | 494,516.6 | 827,408.2 | 68.1 | 1,869.9 | 101.6 | 31.5 | 223.1 | 10.7 | 41.9 | 0.4 | 0.5 | 0.0 | 5.9 | 140.0 | 0.8 | 0.3 | 2.1 | 11.1 |

Values for plasma biomarkers are medians in pg/mL. All median plasma biomarker values were ranked from lowest to highest for each biomarker and coloured from green (lowest) to red (highest).

S2 Table: Multivariate models showing the effect of socioeconomic factors on carotid femoral Pulse Wave Velocity

A. Car Ownership

|  | Fold Change  m/s | 95% Confidence Intervals | p value |
| --- | --- | --- | --- |
| Car Ownership | 1.3 | 1.10 to 1.56 | 0.003 |
| Age (years) | 1.02 | 1.01 to 1.02 | <0.0001 |
| Female gender | 0.82 | 0.74 to 0.92 | 0.001 |
| HIV infection | 1.05 | 0.93 to 1.18 | 0.47 |
| Diastolic Blood Pressure (mmHg) | 1.01 | 1.003 to 1.01 | 0.002 |
| Haemoglobin (g/dL) | 1.01 | 0.98 to 1.04 | 0.41 |

B. Television Ownership

|  | Fold Change  m/s | 95% Confidence Intervals | p value |
| --- | --- | --- | --- |
| Television Ownership | 1.12 | 1.03 to 1.23 | 0.012 |
| Age (years) | 1.02 | 1.01 to 1.02 | <0.0001 |
| Female gender | 0.88 | 0.80 to 0.96 | 0.007 |
| HIV infection | 1.08 | 0.97 to 1.21 | 0.15 |
| Diastolic Blood Pressure (mmHg) | 1.01 | 1.00 to 1.01 | 0.008 |
| Haemoglobin (g/dL) | 1.02 | 1.00 to 1.05 | 0.085 |

C. Electricity supply

|  | Fold Change  %cells | 95% Confidence Intervals | p value |
| --- | --- | --- | --- |
| Electricity supply | 1.09 | 1.01 to 1.17 | 0.029 |
| Age (years) | 1.02 | 1.01 to 1.02 | <0.0001 |
| Female gender | 0.93 | 0.85 to 0.99 | 0.083 |
| HIV infection | 1.12 | 1.02 to 1.23 | 0,02 |
| Diastolic Blood Pressure (mmHg) | 1.01 | 1.00 to 1.01 | <0.0001 |
| Haemoglobin (g/dL) | 1.02 | 1.00 to 1.04 | 0.097 |

S3 Table. Multivariate analysis of the effect of education on inflammatory markers identified through univariate analysis

A. IL7

|  | Fold Change  (µg/ml) | 95% Confidence Intervals | p value |
| --- | --- | --- | --- |
| Some education* | 0.20 | 0.07 to 0.59 | 0.002 |
| Age (years) | -0.10 | -0.33 to 0.13 | 0.40 |
| Female gender | -0.26 | -4.22 to 3.71 | 0.90 |

*Comparing those with some education to those who had none

S4 Table. Multivariate analysis of the effect of income on inflammatory markers identified through univariate analysis

A. %Activated CD8 Tcells

|  | Fold Change  %cells | 95% Confidence Intervals | p value |
| --- | --- | --- | --- |
| Income >25 USD/month* | 12.7 | 7.75 to 17.78 | <0.0001 |
| Age (years) | 0.089 | -0.25 to 0.43 | 0.60 |
| Female gender | 3.7 | -2.2 to 9.6 | 0.22 |

B. %Exhausted CD8 Tcells

|  | Fold Change  %cells | 95% Confidence Intervals | p value |
| --- | --- | --- | --- |
| Income >25 USD/month* | 6.77 | 1.30 to 12.24 | 0.016 |
| Age (years) | -0.35 | -0.72 to 0.019 | 0.66 |
| Female gender | 2.08 | -4.46 to .68 | 0.53 |

C. %Intermediate Monocytes

|  | Fold Change  %cells | 95% Confidence Intervals | p value |
| --- | --- | --- | --- |
| Income >25 USD/month* | -1.91 | -3.91 to 0.10 | 0.063 |
| Age (years) | 0.002 | -0.13 to 0.14 | 0.98 |
| Female gender | 0.72 | -1.6 to 3.0 | 0.54 |

D. %Nonclassical Monocytes

|  | Fold Change  %cells | 95% Confidence Intervals | p value |
| --- | --- | --- | --- |
| Income >25 USD/month* | -5.23 | -8.94 to -1.53 | 0.006 |
| Age (years) | -0.016 | -0.28 to 0.24 | 0.90 |
| Female gender | 2.66 | -1.79 to 7.11 | 0.24 |

E. IL6

|  | Fold Change  (µg/ml) | 95% Confidence Intervals | p value |
| --- | --- | --- | --- |
| Income >25 USD/month* | 2.9 | 1.13 to 7.6 | 0.028 |
| Age (years) | 0.049 | -0.20 to 0.21 | 0.56 |
| Female gender | 1.96 | -0.94 to 4.87 | 0.18 |

F. IL13

|  | Fold Change  (µg/ml) | 95% Confidence Intervals | p value |
| --- | --- | --- | --- |
| Income >25 USD/month* | 3.1 | 1.2 to 8.3 | 0.025 |
| Age (years) | -0.08 | -0.18 to 0.02 | 0.12 |
| Female gender | -0.93 | -2.63 to 0.78 | 0.28 |

*Comparing those with income greater than 25 USD per month to those earning 25 or less

S5 Table. Multivariate analysis of the effect of growing crops at home on inflammatory markers identified through univariate analysis

A. %Activated CD8 Tcells

|  | Fold Change  %cells | 95% Confidence Intervals | p value |
| --- | --- | --- | --- |
| Grows crops at home | 12.1 | 5.2 to 19.0 | 0.001 |
| Age (years) | 0.002 | -0.25 to 0.25 | 0.99 |
| Female gender | 3.30 | -1.22 to 7.82 | 0.15 |

B. IL7

|  | Fold Change  (µg/ml) | 95% Confidence Intervals | p value |
| --- | --- | --- | --- |
| Grows crops at home | 2.47 | 1.10 to 5.52 | 0.028 |
| Age (years) | 0.005 | -0.23 to 0.24 | 0.97 |
| Female gender | -0.35 | -4.39 to 3.69 | 0.87 |

C. IL8

|  | Fold Change  (µg/ml) | 95% Confidence Intervals | p value |
| --- | --- | --- | --- |
| Grows crops at home | 2.97 | 1.10 to 7.97 | 0.031 |
| Age (years) | -0.20 | -1.21 to 0.80 | 0.69 |
| Female gender | -6.78 | -24.4 to 10.86 | 0.45 |

S6 Table. Multivariate analysis of the effect of having an earth floor on inflammatory markers identified through univariate analysis

A. %Nonclassical Monocytes

|  | Fold Change  %cells | 95% Confidence Intervals | p value |
| --- | --- | --- | --- |
| Earth floor* | -1.37 | -8.09 to 5.34 | 0.69 |
| Age (years) | -0.25 | -0.29 to 0.14 | 0.49 |
| Female gender | -2.09 | -6.20 to 2.01 | 0.31 |

*Participants with an earth floor compared to all other types of flooring combined

S7 Table. Multivariate analysis of the effect of water kiosk on inflammatory markers identified through univariate analysis

A. %Activated CD8 Tcells

|  | Fold Change  %cells | 95% Confidence Intervals | p value |
| --- | --- | --- | --- |
| Water Kiosk use* | -7.05 | -12.1 to -1.97 | 0.007 |
| Age (years) | -0.05 | -0.30 to 0.20 | 0.68 |
| Female gender | 3.5 | -1.04 to 8.0 | 0.13 |

B. MIP1β

|  | Fold Change  (µg/ml) | 95% Confidence Intervals | p value |
| --- | --- | --- | --- |
| Water Kiosk use* | 0.63 | 0.40 to 0.99 | 0.047 |
| Age (years) | -0.20 | -1.08 to 0.68 | 0.65 |
| Female gender | -3.8 | -19.4 to 11.7 | 0.63 |

C. sICAM1

|  | Fold Change  (pg/ml) | 95% Confidence Intervals | p value |
| --- | --- | --- | --- |
| Water Kiosk use* | 0.65 | 0.44 to 0.59 | 0.026 |
| Age (years) | -1.40 | -6.91 to 4.12 | 0.62 |
| Female gender | 3.06 | -6.63 to 12.8 | 0.53 |

D. sVCAM1

|  | Fold Change  (pg/ml) | 95% Confidence Intervals | p value |
| --- | --- | --- | --- |
| Water Kiosk use* | 0.74 | 0.57 to 0.97 | 0.031 |
| Age (years) | -2.3 | -8.7 to 4.3 | 0.50 |
| Female gender | 0.03 | -0.3 to 0.4 | 0.13 |

E. IL12p70

|  | Fold Change  (µg/ml) | 95% Confidence Intervals | p value |
| --- | --- | --- | --- |
| Water Kiosk use* | 2.39 | 1.34 to 4.28 | 0.003 |
| Age (years) | -0.002 | -0.003 to 0.003 | 0.83 |
| Female gender | 0.022 | -0.03 to 0.07 | 0.40 |

*Compared to all other water sources combined
