# Supplementary material for: Inflammatory pathways amongst people living with HIV in Malawi differ according to socioeconomic status
Source: PLoS One. 2021 Aug 25;16(8):e0256576. doi: 10.1371/journal.pone.0256576 (PMC8386842; doi:10.1371/journal.pone.0256576)
Supplement: Attachment — Submitted filename: Socioeconomic analysis replies to reviewers.docx [file pone.0256576.s002.docx]

Dr. Christine Kelly

Centre for Experimental Host Pathogen Research (CEPHR)

Belfield

UCD

Dublin

T +353 1 7164552

[christine.kelly@ucd.ie](mailto:christine.kelly@ucd.ie)

**18^th^ April 2021**

Your ref: PONE-D-20-21907

**RE: Inflammatory pathways amongst people living with HIV in Malawi differ according to socioeconomic status**

Dear Professor Uthman,

Please find enclosed responses to the reviewers’ comments for the above manuscript. Please do not hesitate to get in touch of any further clarification is required.

Yours sincerely,

Christine Kelly

(On behalf of co-authors)

Reviewer #1

Abstract:
“Results: Of 279 PLWH, the median (IQR) age was 36 (31-43) years and 122 (44%) female” – should this be were female?

Correction added to manuscript

“and amongst those earning less than 10 USD/month compared to 100-150 USD/month (10.5% versus 8.0%; p=0.08)” I am not sure why this finding is specifically mentioned in the abstract since it was not statistically significant.

Thank you for highlighting this inaccuracy. Correction added to manuscript.

Line 45-7: “Non-communicable diseases (NCDs) are fast becoming the leading cause of mortality in low income sub-Saharan Africa (SSA), with DALYs related to NCDs fast approaching those from including communicable diseases(1).” – Please rephrase for clarity: what does from including communicable diseases mean?

Thank you for highlighting this typographical error. Corrected in manuscript.

Line 50-1: “Persistent inflammation and resultant endothelial damage amongst PLWH is implicated…” – This should be plural.

Corrected in manuscript.

Some abbreviations are not explained e.g. DALYs, TB, PBMCs, IQR.

Abbreviations now explained in the manuscript.

Line 89: Was cfPWV performed by a trained person? Was it performed by a single person? If not, was inter-rater concordance assessed?

Full training for cfPWV was provided by colleagues at the Institute of Cardiovascular Sciences, UCL (included on authorship). A sentence has been added to the manuscript to direct readers to a reference with a more detailed outline of how cfPWV was conducted for this cohort.

Line 90: When was the socio-economic evaluation done? If done at both time points, which values were used?

The assessment was carried out at baseline visit – this has been clarified in the manuscript.

Line 91: Has the standardised questionnaire for assessment of socio-economic status been validated for the sub-Saharan African setting?

The questionnaire has been used in HIV clinical trials across low-income sub-Saharan Africa, and was initially designed for use in the DART trial (*Routine versus clinically driven laboratory monitoring of HIV antiretroviral therapy in Africa (DART): a randomised non-inferiority trial. January 2010. DOI: 10.1016/S0140-6736(09)62067-5*).

Line 97-103: No information is provided about the instrument used for flow cytometry, the antibody manufacturer, the T-cell subsets examined, or the gating strategy used.

A reference has been inserted to refer the reader to the immunophenotyping protocol previously published.

Line 104: “Proinflammatory Panel-1 (IFN-Ɣ, IL-1β, IL-2, IL-4, IL-6, IL-8, IL-10, IL12p70, IL-13, TNF-α)” – IL1Ra, IL-10 and IL-13 are anti-inflammatory markers.

“Proinflammatory Panel 1” is the name of the panel according to the manufacturers (MSD) and has been used here to make it easier for the reader to replicate the panel.

Line 101-6: It would be better to write all the biomarker names out in full the first time they are used, since the abbreviations may not be familiar to all the readership of this journal.

The abbreviations used are standardised cytokine terminology and the authors would be concerned that writing them out in full might make the manuscript difficult to read.

Line 110-1: “CMV viral loads were quantified by DNA PCR in a subset of participants with available plasma as described previously(20).” Please explain who this subset was or what a decision to test for CMV was based on.

This subset consisted of participants with stored plasma available for analysis. This sentence has been changed to provide clarity for the reader.

Write numbers in the beginning of a sentence out in full.

Corrected in the manuscript

Line 137: Were HIV-uninfected participants also screened for CMV infection?

Yes, participants without HIV also had CMV PCRs performed but did not have detectable virus.

Line 138: Was cfPWV only performed for the HIV-positive participants?

cfPWV was performed for all participants but the median for HIV positive patients is reported for context here. The effect of HIV on cfPWV was the subject of an earlier publication which has been referenced.

Line 151: “HIV cohort, who were also more likely to work 7 hours per week…” – should this be 7 days per week?

We thank the reviewer for highlighting this error which has now been corrected.

Table 1: Please check all numbers in Table 1. For instance, the categories for the floor variable do not add up to 100% for the HIV-infected participants.

We thank the reviewer for point out this rounding error – this has been corrected.

In addition, 198/262 does not equal 80%; 71/262 does not equal 29%; 139/262 does not equal 56%; 90/105 does not equal 84%; 52/105 does not equal 51%; 64/105 does not equal 62%; etc, etc. Note that dark lines that divides the “mode of transport” category.

Whilst the total number of observations for the socio-economic questionnaires is given at the top of the table, the denominator for the percentages in the table may change for variables where some data are missing. This denominator can be derived from the percentage.

Line 158-9: “cfPWV was higher for the 16 (8%) participants who travelled to work in a car compared to those who did not [8m/s (7.3 to 10.2)”. It is not clear what is being presented: median and interquartile range? This is also true later in the manuscript e.g. when monocyte data are presented.

This has been clarified in the manuscript.

Line 161: “p0.0005” should be p=0.0005.

Corrected in manuscript.

Line 162: “These factors remained predictive of cfPWV”. It seems that associated is the more appropriate word here since it does not appear as if predictive models had been constructed.

Corrected in manuscript.

If “Carotid femoral pulse wave velocity (cfPWV) was also carried out at both time points as a
measurement of arterial stiffness”, which values are presented in lines 137 and 138, and which were used in the section “Socio-economic predictors of cfPWV amongst PLWH”? Had there been any change in cfPWV over time?

The analyses presented here are baseline cfPWV values, this has been corrected in the manuscript. Where changes over time were associated with socio-economic variables, the relationship has been reported in the relevant results section. For example, second paragraph of ‘Education level and income section’ – “Participants with a tertiary education tended to experience greater improvements in CD8 activation after 42 weeks of ART compared to those without any education [%change -5.8 (-16.7 to 2.2) versus 2.4 (-7.9 to 12); p=0.07], as did those on higher incomes [% change 6.0 (-8.2 to 20.1) versus -18.2 (-21.1 to -4.8) comparing lowest and highest income categories; p value=0.0001]. However, neither educational level nor patient income predicted change in the proportion of intermediate monocytes on ART (p=0.69 and 0.41 respectively).”

Since many readers will not be familiar with cfPWV, there should be some explanation about what higher values mean in terms of arterial stiffness.

An explanatory paragraph on this has been added to the introduction.

Line 174: What is meant by “an indirect correlation”? According to the statistics, no correlations had been performed.

This has been amended in the manuscript.

Line 181: “as did those on higher incomes” – should this be with higher incomes?

Corrected in manuscript

Lines 185-195: What is meant by the fold change values? Is this the change over time? Why was this selected rather than the median values for each time point? This is not explained in the statistical analysis section.

Values here are median change over time between baseline and exit visits, to provide an indication of how the proportion of T cells have changed on ART. This has been clarified in the text.

Line 191: Since IL-6 has been reported to be affected by obesity, was the association with higher income assessed for confounding with body mass index?

For this analysis, the relationship between inflammatory markers and socioeconomic status were adjusted for age and sex. The effect of obesity would be outside the scope of this paper, but would certainly be important to look at in future studies.

Line 191-3: “Levels of IL6 and IL13 were also higher amongst people with higher incomes [2.9 μg/mL (1.13 to 7.6), p=0.028; and 3.1 μg/mL (1.2 to 8.3), p=0.025 respectively].” Why are the median values (I presume these are medians and IQRs?) shown here and not the fold change? Which time point is referred to? Why are the comparator values not shown for participants with lower incomes? Which groups were combined to create the group “higher incomes”?

These values are fold change with 95% CI, in keeping with the values reported in the previous sentence, this has been repeated for clarity. The time point is at baseline, as for all cross-sectional analysis throughout the manuscript. The comparator >25USD per month to <25 USD per month has been made clearer in the text.

Line 193: What is meant by “Adjusted nonclassical and intermediate monocytes”?

This has been clarified in the manuscript.

Line 211-4: “Baseline CD8 activation remained significantly higher amongst those who grew crops in adjusted analysis [fold change 12.1% (5.2 to 213 19.0);p=0.001], with significantly higher adjusted levels of IL7 and IL8 [IL7 fold change 214 2.47pg/mL (95%CI 1.10 to 5.52),p=0.028; IL8 2.97pg/mL (1.10 to 7.97),p=0.031].” Where are comparator values for participants who did not grow crops? The same is true for lines 231-5.

These values are fold change from regression analysis comparing values among those who did grow crops to values for those who did not. A separate comparator group is therefore not presented.

A table with the descriptive results, as well as the unadjusted and adjusted results of the univariate and multivariable analysis of all the biomarkers tested would be very helpful and greatly improve understanding of the results. One would like to see the results of all the markers that had been tested. In addition, results should be displayed in a systematic fashion since now only results that the authors seemingly found interesting are displayed, even in the supplementary tables. This makes a full assessment of the results impossible.

Due to the volume of data it is challenging to present all of the univariate and multivariate analyses together, and therefore we preferred to present those results that were statistically significant, biologically important and relevant to the message of the paper. To help provide clarity we have provided a new table in supplementary data showing median values of plasma biomarkers for each socio-economic variable.

Line 231: “After adjustment for confounders…”. It is not clear what confounding had been adjusted for with regards to CD8 activation.

CD8 activation is adjusted for age and sex, and is outlined in the methods statement as well as the supplementary material.

Had any adjustment been made for CMV co-infection?

Adjustment was not made for CMV as planned multi-variate analysis did not include CMV as a confounder or mediator.

Monocytes were only defined as classical (CD14++CD16-), intermediate (CD14++CD16+) or nonclassical (CD14+CD16+) and no markers of activation had been included in the flow cytometric analysis. For this reason, any statement about monocyte activation is incorrect and should be rephrased and re-interpreted according to the phenotype observed. Just two examples where this is problematic are the following: “population did not show improvements in monocyte activation on ART.”; “Intermediate monocytes were expanded amongst four PLWH who used an unprotected well as their main water source. Although this group is very small, this finding is in keeping with low socio-economic status and unsafe water sources being associated with monocyte activation and innate inflammatory pathways(29).” As it stands, it is completely unclear what it means to have a higher proportion of intermediate monocytes, for instance.

On activation, monocytes express CD16, signally a proinflammatory phenotype. For clarity, the term monocyte activation has been replaced in the manuscript in the two instances that the term was used, as outlined by the reviewer.

It is not clear what the purpose of the control group is, since only socio-economic variables were compared between them and the HIV-infected participants.

The purpose of the control group here was to place socio-economic status for people living with HIV in the context of socio-economic status in Malawi.

Figure 1: there is no indication of what the asterisks mean.

A figure legend has been added to clarify this.

Figure 2 is very difficult to understand and should be augmented with a footnote that describes what is depicted.

A figure legend has been added to explain this.

Figure 4 would be greatly strengthened by the inclusion of supporting references. It is not clear how viral antigen stimulation leads to lymph node fibrosis.

A figure legend has been added to figure 4 with references and a clear explanation that it is a hypothesis which requires further research.

Why were CD4 cell markers not also assessed? Why are the data on T cell exhaustion and senescence not also shown?

Neither CD4 T cell data nor exhaustion / senescence markers demonstrated an association with socio-economic variables and are therefore not reported in line with the methodological approach outlined. It should be noted that for this population with low nadir CD4 count, few CD4 T cell events were captured.

Reviewer #2: 
• What is a water kiosk? The authors report that it provides clean water but I would like a little more information on what it is.

We thank the reviewer for highlighting this. The functioning of a water kiosk has been clarified in the discussion.

• In line 151, should 7 hours be 7 days per week

This has been corrected in the manuscript. We thank the reviewer for raising this inconsistency.

• The authors could include a brief comment on what is known on the effect of repeated social stress on myelopoiesis (e.g. [www.pnas.org/content/110/41/16574](http://www.pnas.org/content/110/41/16574))

We thank the reviewer for this helpful suggestion. This has been added to the discussion.

• I think a paragraph on what is known on SES and NCDs in low-income countries would also benefit the paper – e.g. referencing this article amongst others <https://www.thelancet.com/journals/langlo/article/PIIS2214-109X(17)30054-2/fulltext>

Again, we thank the reviewer for this helpful suggestion and have added this point to the discussion.
