# Supplementary material for: Inflammatory pathways amongst people living with HIV in Malawi differ according to socioeconomic status
Source: PLoS One. 2021 Aug 25;16(8):e0256576. doi: 10.1371/journal.pone.0256576 (PMC8386842; doi:10.1371/journal.pone.0256576)
Supplement: Attachment — Submitted filename: Socioeconomic analysis further replies (1).docx [file pone.0256576.s003.docx]

Dr. Christine Kelly

Centre for Experimental Host Pathogen Research (CEPHR)

Belfield

UCD

Dublin

T +353 1 7164552

[christine.kelly@ucd.ie](mailto:christine.kelly@ucd.ie)

**22^nd^ July 2021**

Your ref: PONE-D-20-21907

**RE: Inflammatory pathways amongst people living with HIV in Malawi differ according to socioeconomic status**

Dear Professor Uthman,

Please find enclosed responses to further comments from the first reviewer for the above manuscript. Please do not hesitate to get in touch of any further clarification is required.

Yours sincerely,

Christine Kelly

(On behalf of co-authors)

Abstract: Please correct the following sentence: “Water kiosk use showed a protective association protective against T cell activation…”
This has been corrected in the abstract and now reads “Water kiosk use showed a protective association against T cell activation”.

Introduction: Please include the abbreviation for carotid femoral pulse wave when the term is used for the first in line 61: “Carotid femoral pulse wave velocity is a gold standard measurement of arterial stiffness and has been validated against clinical outcomes in high-income settings…”. Do not introduce the term in line 95.

This has now been corrected in the manuscript and the abbreviation is introduced at the first mention of the term.

In the previous round of reviews, I have requested the authors to write all the biomarker names out in full the first time they are used, since the abbreviations may not be familiar to all the readership of this journal. The authors had responded that “The abbreviations used are standardised cytokine terminology and the authors would be concerned that writing them out in full might make the manuscript difficult to read.” I do not agree. Since the journal is not an Immunology journal and has a wide readership, the terms have to be explained. In fact, explaining the terms, as I have illustrated below, only adds 5 lines to the manuscript and provides the non-immunologist with better understanding.
CD38/HLADR, PD1 and CD57 expression, respectively. Monocytes were defined as classical (CD14++CD16- ), intermediate (CD14++CD16+ ) or nonclassical (CD14+CD16+). Stored plasma was tested for 22 cytokines: Proinflammatory Panel-1 (IFN-Ɣ, IL-1β, IL-2, IL-4, IL-6, IL-8, IL-10, IL12p70, IL-13, TNF-α), Vascular Injury Panel-2 (SAA, CRP, VCAM-1, ICAM-1), Chemokine Panel-1 (MIP-1β, IP-10, MCP-1), Angiogenesis -Panel-1 (VEGF-A, bFGF) and single analyte assays for IL1R antagonist and IL-7.

VERSUS

Cluster of differentiation (CD)38/Human Leukocyte Antigen – DR isotype (HLADR), programmed death (PD)-1 and CD57 expression, respectively. Monocytes were defined as classical (CD14++CD16- ), intermediate (CD14++CD16+ ) or nonclassical (CD14+CD16+). Stored plasma was tested for 22 cytokines: Proinflammatory Panel-1 (interferon [IFN]-Ɣ, interleukin [IL]-1β, IL-2, IL-4, IL-6, IL-8, IL-10, IL12p70, IL-13, tumour necrosis factor [TNF]-α), Vascular Injury Panel-2 (serum amyloid A [SAA], C-reactive protein [CRP], vascular cell adhesion molecule [VCAM]-1, intercellular adhesion molecule [ICAM-1]), Chemokine Panel-1 (macrophage inflammatory protein [MIP]-1β, interferon γ-induced protein [IP]-10, monocyte chemoattractant protein [MCP]-1), Angiogenesis -Panel-1 (
vascular endothelial growth factor [VEGF]-A, basic fibroblast growth factor [bFGF]) and single analyte assays for IL-1receptor antagonist (IL-1Ra) and IL-7.

We thank the reviewer for their helpful comment and have provided the explanations for the abbreviations as suggested.

As requested previously, please write numbers out in the beginning of sentences, e.g. “15 (4%) participants died during the study”; “201 (55%) had electricity and 95 (26%) had a private water tap at home. 75 (21%) were unemployed and of those employed, 108 (29%) earned less than 25 USD per month, although 97 (36%) of participants were not able to provide an estimate of monthly salary. 339 (92%) of participants felt that their household did not have enough food.”

Numbers at the beginning of sentences have now been written out in full throughout the manuscript. We thank the reviewer for highlighting this.

One of my previous comments was about Table 1, where the percentages were not correct according to the denominator provided. The authors responded as follows: “Whilst the total number of observations for the socio-economic questionnaires is given at the top of the table, the denominator for the percentages in the table may change for variables where some data are missing. This denominator can be derived from the percentage.” It is good scientific practice to indicate where data are missing and the authors should please make these amendments to the table.

The authors recognise that it would be ideal to include the number of observations and/or missing data for each variable in table 1. However, table 1 already contains a lot of data and we feel that including frequency and percentage for each variable communicates the main message. We have added a foot note to explain that there are some missing data and that the number of observations can be calculated from the denominator of the percentage if the reader wishes.

I still have difficulty in understanding this section: “On univariate analysis of the HIV positive cohort, cfPWV was higher for the 16 (8%) participants who travelled to work in a car compared to those who did not [median (IQR) 8m/s (7.3 to 10.2) versus 7.2m/s (6.3 to 8.1); p=0.008]; for those who owned a television [7.7 (6.7 to 8.5) versus 7.2 (6.2 to 7.8) p=0.0005]; and for those who had an electricity supply at home [(7.4 (6.5 to 8.3) versus 7.2 (6.4 to 8.1) p=0.093]. These factors remained associated with cfPWV when adjusted for age, sex, blood pressure and haemoglobin: car ownership [fold change 1.3m/s (95% CI 1.10 to 1.56); p=0.003]; television ownership [1.12m/s (1.03 to 14 174 1.23); p=0.012]; and electricity access [1.09m/s (1.01 to 1.17); p=0.029].” For the univariate analysis, it seems that the baseline cfPWV is used, but for the multivariable analysis, which I assume are the results given after adjusting for age, sex, blood pressure and haemoglobin, the fold change of the cfPWV is given. What is the rationale for that?

Univariate analysis is conducted using Wilcoxon ranksum which produces an estimate of the difference in medians – therefore median cfPWV is presented. Multivariate analysis is conducted using linear regression which produces a fold change – therefore fold change in cfPWV is presented.

It is unclear how the authors decided whether a change in CD8 activation is an improvement or a deterioration: “Participants with a tertiary education tended to experience greater improvements in CD8 activation after 42 weeks of ART compared to those without any education [%median (IQR) change from baseline to 42 weeks -5.8 (-16.7 to 2.2) versus 2.4 (-7.9 to 12); p=0.07], as did those with higher incomes [% change 6.0 (-8.2 to 20.1) versus -18.2 (-21.1 to -4.8) comparing lowest and highest income categories; p value=0.0001].” This is especially true since no control values are shown and there are no reference values of CD8 activation one can refer to. So, while one group can have higher or lower levels, how does one know which one is better?

The purpose of the paper is to link immune activation with socio-economic status. T cell activation is an extremely well documented marker of immune activation in HIV. Comparisons with other cohorts have been dealt with in referenced studies and the authors feel detailed explanations of this would be outside the remit of this current paper. A sentence on the aim of the paper is added to the end of the introduction to make this clearer.

Similarly, how did the researchers decide what is an improvement in monocyte phenotype: “Compared to those with a brick floor, participants with an earth floor had higher proportions of nonclassical monocytes at presentation [mean (IQR) 16.9% (9.9 to 25.80) versus 10.3% (8.2 – 16.7); p=0.04] and were also more likely to experience improvements after 42 weeks of ART [nonclassical monocyte mean (IQR) change -2.2% (-12.0 to 2.9) versus 5.8% (3.9 to 7.2); p=0.026].”

Classical monocytes (CD14++CD16-) are resting monocytes which are stimulated to become intermediate, also known as inflammatory monocytes, (CD14++CD16+) when exposed to antigen. Non-classical monocytes (CD14+CD16+) are less well understood, but have a large body of evidence supporting the fact that they also stem from activation of classical monocytes. The authors feel that a full explanation of the function of monocyte subsets is outside the remit of this paper, but is discussed in referenced studies.

“Adjusting for age and sex, we first compared those with some education to those without any: IL7 was lower for those with some education [fold change 0.20µg/mL (0.07 to 0.59); p=0.002] who also trended towards lower bFGF [fold change 0.22µg/mL (0.048 to 1.09); p=0.056] and higher proportion of activated CD8 T cells [fold change 7.02% (-0.97 to 15.0); p=0.085].” I am very sorry if I am just not understanding this. The authors state that IL-7 was lower, but show the fold change in support. Do they therefore mean that the fold change was greater in this group or do they mean that the exit value was lower? Even after re-reading the statistics section several times, I still fail to understand what the authors are trying to show.

This analysis is conducted at one single timepoint – baseline recruitment. This is the same throughout the manuscript unless otherwise stated. Fold change refers to the change in value between the two groups of comparison following linear regression analysis. So, taking the first sentence here as an example, the adjusted fold change in IL7 in those with some education compared to those without any education was 0.20pg/mL. So those who had some education had an IL-7 which was 0.20 fold lower than those who had no education when analysed in a regression analysis adjusted for the mentioned factors.

“Interestingly, an indirect association was observed with intermediate monocytes, with higher median (IQR) amongst those with no education compared to a tertiary education [12.6% (5.4 – 15.5) versus 7.3% (5.3 – 9.8); p=0.01] …” Do the authors mean an inverse association instead of an indirect association?

“indirect” has been changed to “inverse” in the manuscript as suggested.

A limitations that has to be mentioned is that there was no adjustment for other clinically relevant parameters such as obesity. This is especially important since some of the findings, such as “Levels of IL6 and IL13 were also higher amongst people with higher incomes [fold change (95% CI) 2.9 µg/mL (1.13 to 7.6), p=0.028; and 3.1 µg/mL (1.2 205 to 8.3), p=0.025 respectively]” could be severely confounded by body weight.

Univariate predictors of immune activation were previously investigated for this cohort – which is how the variables that the models have been adjusted for were identified. Please see the methods section: “Regression models examining associations with socioeconomic variables were adjusted *a priori* for age and sex as confounders for immune activation markers and for age, sex, blood pressure and haemoglobin for cfPWV as previously identified (4).” A reference to this paper has been added to the methods section if readers wish to look into this further.

Since non-classical monocytes are associated with a less inflammatory phenotype and also with tissue repair, how do the authors explain the following finding? “Conversely CD16 positive monocytes, both non-classical and intermediate, were expanded amongst those with variables consistent with a lower socio-economic status including lower income, less education and earth flooring. This expansion of CD16 positive monocytes did not resolve on ART.” This is also contradicted in the following statement: “Participants in lower socio-economic groups experience expanded inflammatory monocytes, which did not improve on ART.” While monocyte phenotypes are extraordinarily complex and still a topic of debate, it is generally accepted that classical monocytes (CD14+ CD16-) are the most pro-inflammatory. See for instance Kapellos TS et al. Human Monocyte Subsets and Phenotypes in Major Chronic Inflammatory Diseases. Front. Immunol., 30 August 2019 | <https://doi.org/10.3389/fimmu.2019.02035>

Intermediate and non-classical monocytes are shown to result from classical monocyte activation. The authors recognise that how non-classical monocytes relate to inflammation is yet to be fully elucidated, but because CD16+ expression does result from classical monocyte activation, we have grouped them together in an activated group. Further explanation of how the function of these monocytes relates to socio-economic status is outside the scope of this paper, but would be an interesting subject for future research. The authors have changed ‘inflammatory; to ‘CD16+’ in the manuscript to provide clarity.

It is not clear why CMV was tested for but not adjusted for in the multivariable analysis since CMV is known to significantly affect CD8+ T-cell and monocyte populations, and especially since there was an association in this study with water use, suggesting that some of the findings related to water use could be confounded by CMV. To list but two relevant references in this regard:
van den Berg SPH, Pardieck IN, Lanfermeijer J, et al. The hallmarks of CMV-specific CD8 T-cell differentiation. Med Microbiol Immunol. 2019;208(3-4):365-373. doi:10.1007/s00430-019-00608-7
Baasch S, Ruzsics Z, Henneke P. Cytomegaloviruses and Macrophages—Friends and Foes From Early on? Front. Immunol., 12 May 2020 | <https://doi.org/10.3389/fimmu.2020.00793>
At the very least, this should be stated as a limitation.

CMV PCR positivity was not associated with CD8 T cell activation on univariate analysis in this cohort as previously reported. This is likely due to high seroprevalence rates of CMV in a setting like Malawi, as well as the fact that it was a HIV population with advanced immune suppression and other more important drivers of CD8 T cell activation.

Can the authors hypothesize as to how the following markers can be reconciled, especially since higher levels of IL-12p70 are known to enhance the cytotoxic effects of CD8+ T-cells?
“After adjustment for confounders, CD8 activation remained lower amongst water kiosk users [fold change -7.05% (95%CI -12.1 to -1.97); p=0.007]. MIP1β, sICAM1 and sVCAM1 were also all significantly lower amongst kiosk users [fold change 0.63pg/mL (95%CI 0.40 to 254 0.99), p=0.047; 0.65µg/mL (0.44 to 0.59), p=0.026; and 0.74 µg/mL (0.57 to 0.97), p=0.03 respectively] but IL12p70 was higher [2.39 (1.34 to 4.28); 0=0.003].” Please also correct 0=0.003.

IL12p70 is naturally produced by macrophages and dendritic cells and therefore may be raised due to production by cells that were not quantified within this study. This would also be an interesting area of future research. The correction has been made accordingly.
